# Associations between Fear of COVID-19, Depression, and Internet Addiction in South Korean Adults

**DOI:** 10.3390/healthcare10050861

**Published:** 2022-05-06

**Authors:** Jung Jae Lee, Sun-Hwa Shin

**Affiliations:** 1 School of Nursing, The University of Hong Kong, Hong Kong 070, China; leejay@hku.hk; 2 Nursing Department, College of Nursing, Sahmyook University, Seoul 01795, Korea

**Keywords:** coronavirus disease 2019, internet addiction, fear, depression

## Abstract

This study investigates the associations between Internet addiction and psychological distress, including fear of coronavirus disease 2019 (COVID-19), and depression in South Korean adults during the COVID-19 pandemic. A population-based cross-sectional online survey was conducted from 14 to 18 May 2021. Potential adult survey respondents aged 20 years or older were randomly extracted from one of the largest online survey panels in South Korea, matching the South Korean national demographic proportions (i.e., region, gender, and age). Subsequently, 1155 participants were included in the study. Descriptive and logistic regression analyses were performed to investigate the participants’ characteristics and analyze the adjusted odds ratios (aORs). Of the South Korean adults, 11.17%, 15.15%, and 37.23% were at risk of Internet addiction, COVID-19 fear, and depression, respectively. Internet addiction was positively associated with COVID-19 fear (aOR = 3.25, 95% CI [2.10, 5.04]) and depressive symptoms (aOR = 6.40, 95% CI [4.15, 9.86]). Addictive Internet use was significantly associated with psychological distress during the COVID-19 pandemic. The development of public health strategies that mitigate addictive Internet use and increase eHealth literacy will be useful for securing the public’s psychological well-being.

## 1. Introduction

Coronavirus disease 2019 (COVID-19) is not simply an infectious disease that threatens human health worldwide but has also had widespread economic and societal impacts [1,2]. Responses to the rapid global spread of COVID-19 included the immediate shutdown of local and international communities, drastically changing the way people worked and lived. These changes that occurred almost overnight resulted in psychological distress, such as fear, anger, depression, and anxiety [2,3], while adding to the growing experience of social isolation and disconnection [3,4,5]. As the COVID-19 pandemic continues, the prevalence of psychological distress also continues to rise [3,4,5], and the most significant reason is the fear of infection with COVID-19 [2,6]. A meta-analysis reported that fear of COVID-19 was significantly associated with other psychological distress, such as depression, anxiety, and post-traumatic stress [7]. Fear of COVID-19 was also found to increase loneliness and smartphone addiction, acting as a mediator that directly or indirectly affects psychological distress [8].

Although the Internet has brought several advantages to people’s daily lives, including in the areas of communication and healthcare, excessive use of the Internet has also caused several problems that cannot be overlooked [9,10], such as Internet addiction, which is defined as “excessive or poorly controlled preoccupations, urges or behavior regarding computer use and Internet access that lead to impairment or distress” [11], p. 353. It is well-documented in the literature that Internet addiction is associated with psychological distress, such as depression [12,13,14], anxiety [14,15], loneliness [16,17], and impulsivity [15,18,19]. A meta-analysis identified the mutual effects between psychological distress and Internet addiction, namely, the effect of psychological distress on Internet addiction and the effect of Internet addiction on psychological distress [20].

The benefits of the Internet have become more apparent during the pandemic owing to social distancing measures, as it bridges the gap between people and allows for certain daily life activities, such as online work meetings, online teaching, and social gatherings [3,4,12,21]. Internet use during the COVID-19 pandemic has increased exponentially, with a study reporting that the duration of Internet use had increased by more than 52.0% compared to the pre-COVID-19 period [22]. As the public has spent more time on the Internet during the pandemic, the prevalence of Internet addiction has increased [8,12,23,24]. Therefore, the negative effects of Internet addiction on health, including psychological distress, have become more prevalent during the pandemic, resulting in an emerging public health concern that needs to be addressed [25].

This study investigated the prevalence of psychological distress, including COVID-19 fear and depression, and the association between psychological distress and Internet addiction in South Korean adults. Several studies have explored the association between Internet addiction and psychological distress during the COVID-19 pandemic [12,13,14,15,18,21]. However, these studies recruited only young people (children, adolescents, and university students), while evidence of the association in adults is absent. To the best of our knowledge, this is the first study investigating Internet addiction and psychological distress in adults through a population-based survey in South Korea, where the Internet distribution rate is currently at a high level.

## 2. Materials and Methods

### 2.1. Research Design

A cross-sectional online study was conducted. On the first day of the survey (14 May 2021), there were 130,369 confirmed cases and 1893 deaths in South Korea, with a mortality rate of 1.45%. In addition, the second stage of social distancing measures (Level 2) centered on metropolitan areas in response to the third epidemic, with an average of more than 500 confirmed cases per day [26]. Level 2 social distancing measures in South Korea include the prohibition of private gatherings of more than nine people, bans on large events and gatherings of more than 100 people, restrictions on the operation of multi-use community facilities (e.g., libraries, museums, and movie theaters) after midnight, and, while all schools are open for face-to-face classes, student density is capped at two-thirds of the usual cohort.

### 2.2. Study Participants

The target population was adults aged 20 years or older living in South Korea. Participants were selected if they voluntarily agreed to participate and could read and understand the questionnaire items. This study was commissioned by EMBRAIN (https://www.embrain.com/eng/, accessed on 6 April 2022) (approximately 1.4 million panels in South Korea), one of the largest international survey institutions. Based on the demographics in May 2021, an email invitation to participate was sent to 2151 panel members using the population-proportional allocation randomization method considering the region, gender, and age of adults nationwide. A total of 1337 panel members (response rate: 62.16%) completed the questionnaire. However, if the response time to any section was less than 30 s, or if only one number (i.e., one answer) was selected in the questionnaire, it was considered an insincere response and therefore excluded. Data from 182 participants were excluded from those who completed the questionnaire. Finally, data from 1155 people (response rate 53.69%) were used for the analysis, and the sampling error was ±2.88% *p* at a 95% confidence level.

### 2.3. Instruments

#### 2.3.1. Socio-Demographics

Participant socio-demographics, including age, gender, residential area, monthly income, household arrangements, religion, and occupation, were investigated. Occupation was analyzed by dividing participants into the employed group and the non-employed group. The unemployed group included students (76, 6.6%), housewives (168, 14.5%), and unemployed/retired individuals (120, 10.4%). For COVID-19-related factors, quarantine experience, vaccination status, vaccination confidence, and perceived health during COVID-19 were investigated. For Internet use patterns, changes in the time spent on the Internet before and after the start of the COVID-19 outbreak and the purpose of Internet use were investigated.

#### 2.3.2. Fear of COVID-19

Fear of COVID-19 was measured using a Korean translation of the Fear of COVID-19 Scale [27] developed by Ahorsu et al. [28], targeting university students and adults. The scale consists of seven items, consisting of physiological responses (three items) and emotional responses (four items). The score ranged from 7 to 35, with a higher total score indicating higher fear of COVID-19. Centered on the average of 20.14 points (±standard deviation [SD] 5.18) of COVID-19 fear, +1 SD or more was classified as the high group, and the rest were classified as the low group and analyzed as a dichotomous variable. This was classified into upper and lower groups to facilitate interpretation of the research results. Cronbach’s α of the scale was 0.82 in Ahorsu et al. [28], and 0.88 in the current study.

#### 2.3.3. Depression

Depression was identified using a screening tool from the Patient Health Questionnaire-9 (PHQ-9) developed by Kroenke et al. [29] and adapted and validated as a Korean version by Park et al. [30]. The scores range from 0 to 27, with a higher total score indicating a higher level of depression. The total PHQ-9 score was calculated and interpreted as follows: 0–4 points = no depression, 5–9 points = mild depression, 10–19 points = moderate depression, and 20–27 points = severe depression [29]. A score of 5 or more was classified as the depression group, and a score of ≤4 was assigned to the no-depression group and analyzed as a dichotomous variable. This was classified into a depressed group and a no-depression group to facilitate interpretation of the research results. Cronbach’s α of the screening tool was 0.81 in Park et al. [30], and 0.91 in the current study.

#### 2.3.4. Internet Addiction

Internet addiction was measured using the Internet Addiction Scale developed by the National Information Society Agency (NIA) [31] in South Korea. The scale consists of 15 items. The scores range from 15 to 60, with a higher score indicating a higher tendency toward Internet addiction. The total score and summed scores for each factor were calculated according to the scoring criteria presented by the NIA, and the sum of Factor 1—daily life disorder (No. 1, 5, 6, 10, 13), Factor 3—withdrawal (No. 3, 8, 11, 14), and Factor 4—tolerance (No. 4, 9, 12, 15) were calculated. High-risk users were defined as those with (1) a total score of ≥42, (2) ≥14 for Factor 1, (3) ≥12 for Factor 3, and (4) ≥13 for Factor 4. Potential risk users were classified as those with (1) a total score ranging from 39 to 41 or (2) 13 for Factor 1. General users were classified as those with (1) a total score of ≤38 or (2) a total score that was not in the range of high-risk and potential-risk user groups [31]. Scoring identified 46 high-risk users (3.98%), 83 potential users (7.19%), and 1026 general users (88.83%). The high-risk and potential user groups were analyzed as the at-risk group, and the general user group was analyzed as the not-at-risk group as a dichotomous variable. Cronbach’s α for the Internet Addiction Scale was 0.87.

### 2.4. Data Collection and Ethical Consideration

Before proceeding with data collection, the research procedures and ethical considerations were reviewed by the bioethics committee at the researcher’s affiliated university (IRB No: 2-1040781-A-N-012021028HR). Data were collected from 14 to 18 May 2021. The purpose of this study and the contents and procedures were introduced through a subject explanation, and the participants were informed that they could stop responding to the questionnaire or withdraw consent at any time and that there was no penalty for doing so. If the participant checked the “agree” box on the online questionnaire, they were considered to have given written consent. It was explained that the confidentiality of the collected data was guaranteed, and that all data would be automatically deleted if the investigation was incomplete. In addition, participants were notified that the files were kept in a locked device and were not used for other research purposes or provided to other researchers. A small reward was provided to participants who completed the questionnaire.

### 2.5. Data Analysis

SPSS 25.0 Program (IBM Institute, New York, USA) was used for the data analysis. In the survey, there were no missing data, as it was set up so that participants were not able to move on to the next question if they had not responded to the previous one. The frequency and percentage of the participants’ general, COVID-19-related, and Internet use characteristics were analyzed, and descriptive statistics of COVID-19 fear, depression, and Internet addiction were analyzed. A Pearson’s correlation analysis was performed to examine the relationship between COVID-19 fear, depression, and Internet addiction. The differences between the groups according to general characteristics were analyzed using the χ^2^ test. To determine the effect of COVID-19 fear and depression on the Internet addiction group, a binary logistic regression analysis was performed to analyze the adjusted odds ratio (aOR; adjusted for the participants’ demographics—gender, age, monthly income, household arrangement, religion, and occupation) and 95% confidence interval (CI). All statistical significance levels were set at *p* < 0.05.

## 3. Results

### 3.1. Participants’ General and Internet Usage Characteristics

The general characteristics of the participants are presented in Table 1. The majority (342, 29.61%) were from Gyeonggi-do/Incheon, followed by Gyeongsang-do (296, 25.63%), Seoul (204, 17.66%), Chungcheong-do (136, 11.77%), Jeolla-do (118, 10.22%), and Gangwon-do/Jeju-do (59, 5.11%). There were 587 males (50.82%) and 568 females (49.18%) with an average age of 44.57 years (±13.44). A total of 212 participants were in their 20s (18.35%), 214 in their 30s (18.53%), 251 in their 40s (21.73%), 262 in their 50s (22.68%), and 216 in their 60s (18.70%). A total of 590 participants (51.08%) had a monthly income of KRW 3 million or more, and 989 (85.63%) lived with family members or others, accounting for the majority. There were 664 (57.49%) non-religious participants. The number of participants with a job was 791 (68.48%) of the 1155. A total of 187 participants (16.19%) were subject to self-isolation due to COVID-19-related characteristics, and 51 (4.42%) were vaccinated against COVID-19. A total of 456 (39.48%) participants indicated that they had confidence in the COVID-19 vaccination. The most common perceived health status during the COVID-19 period was neutral (558, 48.31%).

In terms of psychological distress, the average scores for fear of COVID-19 and depression were 20.14 (±5.18) and 4.38 (±4.84), respectively. The average score for Internet addiction was 27.11 (±7.40), and the number of participants in the risk group for Internet addiction was 129 (11.17%).

As a characteristic of Internet use, the average daily usage time before COVID-19 was 3.48 h (±1.98), whereas it was 4.07 h (±2.18) after the outbreak. A total of 455 (39.39%) participants indicated they increased that their Internet use compared to before the COVID-19 outbreak, and 641 (55.50%) answered that the purpose of using the Internet was for information acquisition (search/study/work).

### 3.2. Relationship between COVID-19 Fear, Depression, and Internet Addiction

The correlations between COVID-19 fear, depression, and Internet addiction are presented in Table 2. COVID-19 fear showed a significant positive correlation with depression (r = 0.41, *p* < 0.001; moderate correlation) and Internet addiction (r = 0.24, *p* < 0.001; weak correlation). Depression showed a significant positive correlation with Internet addiction (r = 0.50, *p* < 0.001; moderate correlation).

### 3.3. Differences in COVID-19 Fear, Depression, and Internet Addiction Groups by Participants’ Characteristics

For fear of COVID-19, there were 175 (15.15%, 95% CI = 12.27–18.03%) participants in the high group and 980 (84.85%) participants in the low group. A total of 430 participants (37.23%, 95% CI = 33.35–40.11%) were classified into the depressed group, and the normal group comprised 725 (62.77%) participants. Internet addiction was observed in 129 participants (11.17%, 95% CI = 8.29–14.05%) in the at-risk group and 1026 (88.83%) in the not-at-risk group.

The results of the analysis of the group differences in Internet addiction according to the characteristics of the participants are reported in Table 3. Internet addiction was associated with age (χ^2^ = 28.04, *p* < 0.001), religion (χ^2^ = 3.46, *p* = 0.038), vaccination confidence (χ^2^ = 15.59, *p* < 0.001), perceived health status (χ^2^ = 7.64, *p* = 0.022), Internet use time (χ^2^ = 7.35, *p* = 0.005), and purpose of Internet use (χ^2^ = 12.80, *p* = 0.002).

### 3.4. Association between COVID-19 Fear, Depression, and Internet Addiction

The results of the adjusted odds ratio (aOR) and 95% CI through the logistic regression analysis to examine the effect of COVID-19 fear and depression on Internet addiction are presented in Table 4. After adjusting for demographic characteristics, variables related to COVID-19 and psychological distress were included. Regarding perceived health status during the COVID-19 period, the odds ratio of the unhealthy group belonging to the at-risk group for Internet addiction was 2.13 times higher than that of the healthy group (aOR = 2.13, 95% CI [1.25, 3.62]). In the high group for COVID-19 fear, the odds ratio was 3.25 times higher than in the low group (aOR = 3.25, 95% CI [2.10, 5.04]). In the depressed group, the odds ratio was 6.40 times higher than that in the non-depressed group (aOR = 6.40, 95% CI [4.15, 9.86]).

After constructing four clusters considering COVID-19 fear and depression, the group with low COVID-19 fear and depression was designated as a reference variable for the group at risk of Internet addiction. The odds ratio was analyzed (Table 5). Based on the reference variable, the group with high COVID-19 fear and depression had a 10.22 times higher odds ratio of belonging to the group not at risk of Internet addiction (aOR = 10.22, 95% CI [5.88, 17.77]). This odds ratio was 5.20 times higher in the group with low COVID-19 fear and high depression (aOR = 5.20, 95% CI [3.24, 8.36]).

## 4. Discussion

This study examined the level of COVID-19 fear and depression among South Korean adults when restrictions were prolonged during the COVID-19 pandemic. In the context of heightened Internet use secondary to social distancing rules, the tendency towards Internet addiction was investigated, and factors affecting the Internet addiction group were identified.

Of the participants, 39.39% reported increased time spent on the Internet since the start of the pandemic. This increase is not unexpected, as social distancing has consistently been the main global health advice since the start of the COVID-19 pandemic, prompting stay-at-home advice and work-from-home arrangements across all sectors [12,23]. Indeed, more than half of the participants (55.50%) reported that the main purposes of Internet use were education, work, and information acquisition, which included intensified online searches for COVID-19 information due to the uncertainty surrounding the disease and the future, as well as profound fear of COVID-19 infection [16,21]. The remaining participants reported Internet use purposes such as communication and entertainment. Internet use has risen in an attempt to fill the gap of loneliness caused by pandemic-associated social isolation. Unfortunately, evidence has demonstrated that there is a close relationship between increased Internet use and Internet addiction [16,21]. Notably, in this study, the at-risk group for Internet addiction had a higher rate of Internet use to obtain pleasure through social media, communication, gaming, shopping, and video content. Previous studies have also warned that social isolation and disconnection due to COVID-19 may precipitate unhealthy addiction behaviors to online games, pornography, and gambling in an attempt to replace direct human interaction [32,33]. As such, increased time spent at home due to the COVID-19 pandemic restrictions can lead to excessive Internet use, precipitating the risk of Internet addiction. Additionally, according to a study [34], excessive Internet use increases the risk of exposure to COVID-19-related misinformation, and such exposure has a positive association with psychological distress, such as depression, anxiety, and PTSD symptoms, and negative effects on COVID-19-preventive behavioral performance.

Of all the participants, 15.15% were classified into the high-COVID-19-fear group. While such fear can also increase desirable behaviors to prevent infection, excessive fear can negatively affect an individual’s emotions and behaviors, adversely affecting psychological distress and causing greater anxiety and fear [2,8]. In a previous study, those who answered “I am afraid that life after COVID-19 is unpredictable” were four times more likely to belong to the depressed group, suggesting that fear of COVID-19 increases depression [2]. This study also reported a high prevalence of depressive symptoms (37.23%). As the COVID-19 pandemic continues, it has been recognized that restrictions in daily life routines can result in psychological discomfort such as depression [2,35]. Multiple studies worldwide have reported increased psychological distress due to a protracted pandemic [36,37,38]. A meta-analysis based on studies conducted during the COVID-19 outbreak revealed a depression prevalence of 25.0%, which was more than seven times higher than the global prevalence of 3.44% in 2017 [35]. While social distancing and work-from-home arrangements are likely to remain at varying degrees as the world moves towards a “new normal”, efforts in the future need to be directed towards provided clear and precise public health information about the disease and advice and enhancing eHealth literacy across all age groups to prevent exposure to misinformation and therefore fear.

The current study found that Internet addiction was positively associated with COVID-19 fear (aOR = 3.25, 95% CI [2.10, 5.04]). Furthermore, this study also reported that Internet addiction was significantly associated with depressive symptoms (aOR = 6.40, 95% CI [4.15, 9.86]). These findings correspond with a study reporting that Internet addiction is associated with COVID-19 fear [8,39] and depression [13,36]. Study participants with high COVID-19 fear and depression were ten times more likely to belong to the Internet addiction risk group, resulting in great significance because no previous studies have performed a cluster analysis based on COVID-19 fear and depression. The group with high depression and high COVID-19 fear was associated with Internet addiction, whereas the group with high COVID-19 fear but low depression was not associated with Internet addiction. These findings correlate with another study that revealed that increased time spent on games, novel media, and the Internet was related to depression but not COVID-19 fear [40]. Although this study suggested a relationship between the psychological distress of COVID-19 fear and depression affecting the risk of Internet addiction, it is also necessary to consider the inverse causal relationship in which excessive Internet use aggravates psychological distress [41]. Therefore, it is necessary to conduct a study to confirm the direction of psychological distress and Internet addiction through longitudinal and experimental approaches.

If fear and depression persist due to the prolonged COVID-19 pandemic, the risk of family conflict, violence, and suicidal behavior could increase [42]. In particular, the correlation between Internet use and self-harm/suicidal behavior is highly correlated with Internet addiction behavior [43]. Therefore, it is necessary to focus on public health education to raise awareness of the potentiality of Internet addiction behaviors and offer strategies for people to cope with changes in their social routines while also addressing the psychological distress of fear and depression early on. Prevention of Internet addiction behaviors can assist in reducing the prevalence of psychological distress in the community during the pandemic, while providing preemptive counseling and psychological support services to vulnerable individuals can prevent serious psychological health issues such as self-harm. Public health institutions need to intensively manage psychological distress in times of turbulence caused by infectious diseases and take active measures to prevent the risk of Internet addiction by developing and delivering effective public health interventions that mitigate addictive Internet use and increase eHealth literacy.

Despite the high prevalence of depressive symptoms, as the COVID-19 vaccination rate increases, the hope of returning to one’s previous daily life and routine is increasing [44], which may serve as an opportunity to reduce depression. The depression score of the group with confidence in COVID-19 vaccines was lower than that of the group without confidence. Hence, it is considered that active preventive measures against COVID-19 infection, such as vaccinations, can indirectly manage the emotions of depression.

However, the ratio of high COVID-19 fear was greater in the religious group. This finding differed from other studies that found that religiousness was not related to fear of COVID-19 during the pandemic [45], as it reflects the special situation in South Korea, where prejudice had been formed [46]. As such, the COVID-19 pandemic has affected religion and faith in various ways, creating a situation of tension, conflict, and necessary cooperation between the government and religious groups, especially regarding social distancing guidelines and religious gatherings [47]. COVID-19 fear is closely associated with a wide range of psychological distress [7,8], and reinforced faith is associated with greater psychological distress [45,47]. In addition, it has been reported that adults with a high religious affiliation have strong behavioral intentions to follow COVID-19 guidelines [48]. As places of worship are one of the most affected by COVID-19 restrictions, there should be clear guidelines and strategies to guide and support them in reaching out to their congregation remotely, such as Zoom church services, and to provide ongoing spiritual support through a continued sense of community.

This study has several limitations. First, the participants comprised a panel belonging to a survey institution; therefore, it is necessary to consider limitations when generalizing the research results. Different sampling methods that can generalize the research results to the entire population should be sought in future studies by recruiting various survey companies or seeking a survey method that includes subjects who did not participate as a panel (visits or phone surveys, etc.). Second, because the research data were collected via a self-reported questionnaire, there may be a general selection bias, such as memory recall and desirable social responses. It is necessary to overcome those limitations by considering the use of qualitative data. Third, since this study used a cross-sectional design, and there is a limit to confirming the causal relationship between variables, it is necessary to check the causal relationship through future experiments and longitudinal studies.

## 5. Conclusions

This study identified that South Korean adults who participated in this study were at risk of Internet addiction and psychological distress, including COVID-19 fear and depression, during the COVID-19 pandemic. The factors influencing the Internet addiction risk group were the degree of confidence in vaccination, high COVID-19 fear, and high COVID-19 depression. To prevent the risk of Internet addiction during a large-scale disease epidemic such as COVID-19, it is necessary to manage public fear and uncertainty through transparent information disclosure, and it is confirmed that it is necessary to carefully take care of the mental health of those with depression.

## Figures and Tables

**Table 1:** General characteristics of participants (*N* = 1155).

Characteristics	Variables	Categories	*n* (%)	Mean ± SD
Socio- demographics	Residential area	Seoul	204 (17.66)	
		Gyeonggi-do/Incheon	342 (29.61)	
		Chungcheong-do	136 (11.77)	
		Gyeongsang-do	296 (25.63)	
		Jeolla-do	118 (10.22)	
		Gangwon-do/Jeju-do	59 (5.11)	
	Gender	Male	587 (50.82)	
		Female	568 (49.18)	
	Age (year)	20–29	212 (18.35)	44.57 ± 13.44
		30–39	214 (18.53)	
		40–49	251 (21.73)	
		50–59	262 (22.68)	
		60–69	216 (18.70)	
	Monthly income	<KRW 3,000,000	590 (51.08)	
		≥KRW 3,000,000	565 (48.92)	
	Household arrangement	Living alone	166 (14.37)	
		Living with others	989 (85.63)	
	Religion	None	664 (57.49)	
		Yes	491 (42.51)	
	Occupation	Unemployed	364 (31.52)	
		Employed	791 (68.48)	
COVID-19- related factors	Quarantine experience	No	968 (83.81)	
		Yes	187 (16.19)	
	Vaccination status	No	1104 (95.58)	
		Vaccinated	51 (4.42)	
	Trust in vaccination	Do not trust	285 (24.68)	
		Neutral	414 (35.84)	
		Trust	456 (39.48)	
	Perceived health during COVID-19	Unhealthy	186 (16.10)	
		Neutral	558 (48.31)	
		Healthy	411 (35.58)	
Psychological distress	Fear of COVID-19	Low	980 (84.85)	20.14 ± 5.18
		High	175 (15.15)	
	Depression	None	725 (62.77)	4.38 ± 4.84
		Depressive	430 (37.23)	
Internet use	Internet addiction	Not-at-risk	1026 (88.83)	27.11 ± 7.40
		At-risk	129 (11.17)	
	Changes in time spent on the Internet after COVID-19	Similar or decreased	700 (60.61)	
		Increased	455 (39.39)	
	Purpose of Internet use	Communication	286 (24.76)	
		Entertainment	228 (19.74)	
		Information acquisition	641 (55.50)	

**Table 2:** Correlations between fear of COVID-19, depression, and internet addiction (N = 1155).

Variables	Fear of COVID-19	Depression
Depression	0.41 ***	
Internet addiction	0.24 ***	0.50 ***

*** *p* < 0.001.

**Table 3:** Internet addiction according to participants’ characteristics (N = 1155).

Variables	Categories	Internet Addiction		
		Not-at-Risk Group (*n* = 1026)	At-Risk Group (*n* = 129)	χ^2^
Gender	Male	523 (89.10)	64 (10.90)	0.09
	Female	503 (88.56)	65 (11.44)	
Age (year)	20–29	175 (82.55)	37 (17.45)	28.04 ***
	30–39	180 (84.11)	34 (15.89)	
	40–49	222 (88.45)	29 (11.55)	
	50–59	241 (92.98)	21 (8.02)	
	60–69	208 (96.30)	8 (3.70)	
Monthly income	<KRW 3,000,000	516 (87.46)	74 (12.54)	2.29
	≥KRW 3,000,000	510 (90.27)	55 (9.73)	
Household arrangement	Living alone	143 (86.14)	23 (13.86)	1.41
	Living with others	883 (89.28)	106 (10.72)	
Religion	None	580 (87.35)	84 (12.65)	3.46 *
	Yes	446 (90.84)	45 (9.16)	
Occupation	Unemployed	320 (87.91)	44 (12.09)	0.45
	Employed	706 (89.25)	85 (10.75)	
Quarantine experience	No	864 (89.26)	104 (10.74)	1.09
	Yes	162 (86.63)	25 (13.37)	
Vaccination status	No	45 (88.24)	6 (11.76)	0.02
	Vaccinated	981 (88.86)	123 (11.14)	
Trust in vaccination	Do not trust	235 (82.46)	50 (17.54)	15.59 ***
	Neutral	375 (90.58)	39 (9.42)	
	Trust	416 (91.23)	40 (8.77)	
Perceived health during COVID-19	Unhealthy	155 (83.33)	31 (16.67)	7.64 *
	Neutral	497 (89.07)	61 (10.93)	
	Healthy	374 (91.00)	37 (9.00)	
Changes in time spent on the Internet after COVID-19	Similar or decreased	636 (90.86)	64 (9.14)	7.35 **
	Increased	390 (85.71)	65 (14.29)	
Purpose of Internet use	Communication	390 (85.71)	65 (14.29)	12.80 **
	Entertainment	197 (86.40)	31 (13.60)	
	Information acquisition	588 (91.73)	53 (8.27)	

* *p* < 0.05, ** *p* < 0.01, *** *p* < 0.001.

**Table 4:** Logistic regression of Internet addiction for COVID-19-related factors, Internet use, and psychological distress (N = 1155).

Variables	Categories	Internet Addiction (Not-at-Risk Group/At-Risk Group)	
		Odds Ratio [95% CI]	
		Crude	Adjusted
Quarantined (including family)	No	Reference	Reference
	Yes	1.28 [0.80, 2.05]	1.16 [0.72, 1.88]
Vaccination status	No	Reference	Reference
	Vaccinated	0.94 [0.39, 2.25]	0.85 [0.35, 2.07]
Vaccine trust	Do not trust or uncertain	Reference	Reference
	Trust	0.66 [0.45, 0.98] *	0.84 [0.56, 1.27]
Perceived health during COVID-19	Healthy	Reference	Reference
	Neutral	1.24 [0.81, 1.91]	1.31 [0.85, 2.03]
	Unhealthy	2.02 [1.21, 3.38] **	2.13 [1.25, 3.62] **
Changes in time spent on the Internet after COVID-19	Similar or decreased	Reference	Reference
	Increased	1.66 [1.15, 2.39] **	1.45 [0.99, 2.12]
Purpose of Internet use	Entertainment	Reference	Reference
	Communication	1.49 [0.72, 1.95]	1.05 [0.63, 1.75]
	Information acquisition	0.57 [0.36, 0.92] *	0.70 [0.43, 1.15]
Fear of COVID-19	Low	Reference	Reference
	High	2.71 [1.78, 4.12] ***	3.25 [2.10, 5.04] ***
Depression	None	Reference	Reference
	Depressed	6.61 [4.32, 10.10] **	6.40 [4.15, 9.86] ***

Adjusted for gender, age, monthly income, household arrangement, religion, and occupation; CI = confidence interval; * *p* < 0.05, ** *p* < 0.01, *** *p* < 0.001.

**Table 5:** Logistic regression of Internet addiction for combined psychological distresses (fear of COVID-19 and depression)**.**

Variables	*n*	Internet Addiction (Not-at-Risk Group/At-Risk Group)	
		Odds Ratio [95% CI]	
		Crude	Adjusted
Low fear and No depression	675	Reference	Reference
High fear and No depression	50	0.93 [0.22, 4.01]	1.07 [0.24, 4.65]
Low fear and Depressed	305	5.68 [3.57, 9.05] ***	5.20 [3.24, 8.36] ***
High fear and Depressed	125	9.01 [5.27, 15.41] ***	10.22 [5.88, 17.77] ***

Adjusted for gender, age, monthly income, household arrangement, religion, and occupation; CI = confidence interval; *** *p* < 0.001.

## Data Availability

The data presented in this study are available upon request from the authors.
